# The Role of Virtual Reality in Improving Health Outcomes for Community-Dwelling Older Adults: Systematic Review

**DOI:** 10.2196/17331

**Published:** 2020-06-01

**Authors:** Gordana Dermody, Lisa Whitehead, Graham Wilson, Courtney Glass

**Affiliations:** 1 School of Nursing and Midwifery Edith Cowan University Joondalup Australia; 2 University of Strathclyde Glasgow United Kingdom

**Keywords:** virtual reality, aged, aged, 80 and over, outcome assessment, health care, independent living, systematic review

## Abstract

**Background:**

Virtual reality (VR) delivered through immersive headsets creates an opportunity to deliver interventions to improve physical, mental, and psychosocial health outcomes. VR app studies with older adults have primarily focused on rehabilitation and physical function including gait, balance, fall prevention, pain management, and cognition. Several systematic reviews have previously been conducted, but much of the extant literature is focused on rehabilitation or other institutional settings, and little is known about the effectiveness of VR apps using immersive headsets to target health outcomes among community-dwelling older adults.

**Objective:**

The objective of this review was to evaluate the effectiveness of VR apps delivered using commercially available immersive headsets to improve physical, mental, or psychosocial health outcomes in community-dwelling older adults.

**Methods:**

Peer-reviewed publications that included community-dwelling older adults aged ≥60 years residing in residential aged care settings and nursing homes were included. This systematic review was conducted in accordance with the Joanna Briggs Institute (JBI) methodology for systematic reviews of effectiveness evidence. The title of this review was registered with JBI, and the systematic review protocol was registered with the International Prospective Register of Systematic Reviews.

**Results:**

In total, 7 studies that specifically included community-dwelling older adults were included in this review. VR apps using a head-mounted display led to improvements in a number of health outcomes, including pain management, posture, cognitive functioning specifically related to Alzheimer disease, and a decreased risk of falls. A total of 6 studies reported a statistically significant difference post VR intervention, and 1 study reported an improvement in cognitive function to reduce navigational errors. Only one study reported on the usability and acceptability of the interventions delivered through VR. While one study used a distraction mechanism for pain management, none of the studies used gaming technology to promote enjoyment.

**Conclusions:**

Interventions to improve health outcomes through VR have demonstrated potential; however, the ability to synthesize findings by primary outcome for the older adult population is not possible. A number of factors, especially related to frailty, usability, and acceptability, also need to be explored before more substantial recommendations on the effectiveness of VR interventions for older adults can be made.

**Trial Registration:**

PROSPERO CRD42019143504; https://www.crd.york.ac.uk/prospero/display_record.php?RecordID=143504

## Introduction

### Background

Understanding how to best support the health and well-being of older adults is an important societal question. With increasing age, natural age-related physical, cognitive, and social changes are often compounded by physical and cognitive comorbidities [1-3]. A primary desire of older people as they age is that they are able to remain living as independently as possible [1-3]. However, the majority of older people live with at least one chronic disease with subsequent impact on physical, mental, or psychosocial health [1-3]. This can impact the ability to live independently or maintain social connectivity [1-3]. Prominent musculoskeletal changes include muscle loss and weakness, which impact gait and balance, increasing the risk of falls [4]. Falls among older adults is a global health issue affecting nearly 35% of people aged 65 years and older every year [5]. As age increases, so does the prevalence of falls [6]. Falls are costly to the health care systems and usually impact the quality of life (QoL) and independence of the older person [7]. Psychological and behavioral responses are common in people who have sustained a fall [8]. In addition, normal neurocognitive changes that occur in healthy aging can impact processing speed and motor responses, potentially increasing the risk of falls [9].

Changes in neurocognitive function are prevalent among older adults. Worldwide, 50 million people are diagnosed with dementia, and Alzheimer disease (AD) is the most common form of dementia, contributing nearly 70% to the dementia diagnoses [10]. The brain changes that accompany AD result in memory loss that can disrupt a person’s daily life [11], for example, difficulty in solving problems and completing everyday tasks at home, misplacing things, and getting lost outside the home, which can have a significant impact on the ability to navigate daily life. Frequently, people who experience memory problems withdraw from social activities because the changes they have experienced impact social interaction [12]. Furthermore, the QoL of older adults with dementia may be adversely affected by chronic pain, which is associated with depression [13]. As people with dementia such as AD may have vision and perception problems, they are at a greater risk of sustaining a fall and three times more likely to suffer from injuries such as hip fracture [14]. Interventions to facilitate healthy aging, adaptation, and strengthening in older adults with normal age-related changes that are intertwined with chronic disease are important in working to support older adults to remain in their own homes for as long as possible and to promote well-being. Exploring interventions that use virtual reality (VR) may offer unique opportunities to address areas of health need. VR is evolving at a rapid rate and presents an opportunity to enhance and support older adults’ physical and cognitive issues to promote engagement in physical activity, travel, or interactions with others [15]. The term *virtual reality* was first coined in the late 1980s. In the last 30 years, a vast range of VR devices have been developed, and the technology has been used across various domains, including education, athlete training, architecture, and notably in health-related research including physical, mental, and psychosocial health [15].

The term *virtual reality* has been used in health research to refer to various forms of onscreen digital representation of real objects or environments, such as showing a silhouette of an individual on a TV screen that mimics their physical rehabilitation movements [16]. However, VR is now predominantly used to refer to what was previously qualified as *immersive virtual reality*, which is understood to be viewing a stereoscopic virtual environment through a motion-tracked head-mounted display (HMD) [16]. A stereoscopic display encompasses the field of view and is able to track the motion of the head [16]. VR using an HMD not only provides the user with a particular artificial environment to view but also allows the user to engage with the virtual environment as they can look around by moving the head and by moving around the virtual environment by moving the body [16]. Definitions of what constitutes *immersion* both within and outside VR research vary, but a concept that arises from motion-tracked VR is *presence*, also known as an illusory experience of being in another place [16]. Deeper conceptualizations of presence include illusions of *place* or being in another location and *plausibility*, where events viewed by a person in VR are convincing [17]. Within the virtual world, the events seen within it are felt as if the user is really there or the events are actually taking place [18]. By allowing the user to interact with the VR environment naturally (eg, walking up to it and using a hand to pick up objects), the experience appears real for the user [18]. These attributes make a number of VR apps such as exposure therapy treatment for anxiety highly effective [18].

The degree of *presence* (or place illusion) that a user experiences is largely dependent on the characteristics of the VR technology being used [17,19]. Earlier VR research utilized HMDs with poor immersive qualities and limited technical capabilities, such as a lack of head-tracking, a small field of view, or the use of transparent displays (something closer to augmented reality) [19]. Greater presence is felt when the VR device has a stereoscopic display, has a wider field of view, and has greater amounts of tracked user movement [19]. Real-world health settings are likely to use these commercial headsets, as they are relatively affordable, easy to set up, and they support VR apps development through software such as Unity or Unreal Engine [20,21]. Although nonimmersive systems are interesting and beneficial, many community-dwelling older adults with natural age-related changes may not have the mental and physical capacity that may be needed in a nonimmersive environment.

Immersive headsets, or those that incorporate motion-tracked stereoscopic HMDs, and potentially motion controllers are known to induce a stronger sense of presence and potentially a sense of realism, embodiment, memory, and spatial understanding than nonimmersive devices [17,19,22,23]. Older adults have natural age-related changes that impact cognitive function; the literature discusses that increasing the immersive properties can be beneficial to increasing the sense of presence and place illusion, which is critical to achieve high intrinsic motivation to participate in the VR experience and achieve the health benefits desired [17,24].

Some community-dwelling older adults with cognitive and physical changes may find it challenging to concentrate on images on a wall-mounted flat screen or a handheld tablet while using an exercise bike. Furthermore, spatial cognition and ability are common changes that occur as a result of aging [25]. Given these issues, the participant may experience increased levels of stress during the intervention, and the sense of presence may be diminished as the participant tries to cognitively and physically navigate the intervention [17,24,25]. The task environment (flat screen, computer screen, and tablet) may have several display elements including passive crystal displays and light-emitting diodes [25]. Lighting levels for reading passive liquid crystal displays (nonbacklit) may be adequate for office or research environments; however, they are not adequate when used with older adults who may have visual acuity changes and who will be using the VR HMD in the home environment [25].

Using nonimmersive displays is further compounded by the angle from which the participant may need to view the display and potentially the glare of the screens that are used, which can alter the ability to visualize [25]. Due to these factors, immersive headsets are considered a distinct category of device with unique potential benefits for community-dwelling older adults. Accordingly, VR experiences in health apps for community-dwelling older adults using HMDs with stereoscopic displays that encompass the field of view and track the motion of at least the head, but also additional parts of the body, were examined in this systematic review.

Therefore, a number of papers have evaluated or systematically reviewed literature based on *virtual reality* in the health of older adults; however, as discussed earlier, the definition of *virtual reality* varies widely. The majority of these papers focused on nonimmersive digital games technology such as the Nintendo Wii, Microsoft Kinect, or custom activities where player movement is shown on a flat TV or monitor screen. Common topics of inquiry into this form of *virtual reality* used with older adults include one or more aspects of physical activity such as balance, rehabilitation, gait function, and falls [26-36]. Serious mental illness [37] and cognitive intervention and training [27,38-40] have also been the focus of reviews; however, very few papers have examined immersive VR, the area of interest in this paper. Duque [41] reported using *3D virtual-reality goggles* for visual-vestibular balance training exercises, although it is not clear if these constitute an immersive display or headset. However, recent research has begun to use immersive HMDs in the assessment of physical [42,43] or cognitive function [44] in older adults as well as providing enjoyable travel experiences [45].

In addition to the effectiveness of VR and digital games on chosen outcomes, it is important to understand the usability and acceptability of technology. When a technology such as VR is used, researchers need to be aware that older adults may not be familiar with the technology or they may have unique aging-related physical or cognitive changes that could make participation difficult. Silva [46] conducted a systematic review to identify the study characteristics of VR research with older adults and how VR impacted older adult end users. Silva [46] concluded that design characteristics could be modified to aid older adult users’ experience in using the VR apps. A further systematic review [33] reported evidence on the effectiveness and feasibility of using digital gaming systems within older adults to enable physical activity is weak, with a high risk of bias. Although the authors use the term *virtual reality*, none of the studies used immersive VR HMDs, only traditional flat screen gaming technologies or augmented reality glasses (a transparent display with digital overlay). The acceptance of HMD VR, possible cybersickness, and attitudes toward technology have also been investigated [47]. There was no evidence to suggest that negative attitudes toward VR apps or cybersickness would be barriers to using VR apps with older adults.

### Objectives

Although research using VR and immersive headsets is increasing, more knowledge is needed about the types of VR apps using immersive headsets, the health outcomes targeted, outcomes, and transferability to real-world settings for community-dwelling older adults. The aim of this systematic review was to evaluate the effectiveness of VR apps delivered using commercially available immersive headsets in improving physical, mental, and psychosocial health outcomes in community-dwelling older adults.

## Methods

### Inclusion Criteria

#### Types of Participants

This review included specifically community-dwelling older adults aged 60 years or older. Initial searches of the existing literature demonstrated that the classification of what constituted *older adult* varied. Accordingly, the United Nations definition of older populations as 60 years and over was used because this classification provided a more realistic representation of older adults across different countries with varying life expectancies [48]. Older adults residing in residential aged care settings and nursing homes were included, and hospitalized older adults were excluded from the review. Studies that included participants aged under 60 years in the control group were also included.

#### Interventions

Studies that evaluated the use of VR apps delivered via immersive headsets including a combination of VR delivery, employing immersive headsets with incorporation of projector screens to create other forms of immersive VR experiences, were included in this review. Accordingly, commercial headsets including Rift or Vive and older models that encompass the field of view and have motion tracking were included. Studies that did not use VR headsets were excluded.

#### Comparators

This review considered studies that compared the intervention with conventional therapy and studies that did not use comparisons such as case studies.

#### Outcomes

This review considered studies that included the following outcomes: effectiveness of the VR apps intervention via immersive headset using physical, mental, or psychosocial outcome measures. These outcomes were measured using kinematic and kinetic computer apps and/or statistical software as well as Likert-based questionnaires.

#### Types of Studies

This review considered both experimental and quasi-experimental study designs, including, but not limited to, randomized controlled trials (RCTs), nonrandomized controlled trials, before and after studies, and interrupted time-series studies. In addition, analytical observational studies including prospective and retrospective cohort studies, case-control studies, and analytical cross-sectional studies were considered for inclusion. This review also considered descriptive observational study designs including case series, individual case reports, and descriptive cross-sectional studies for inclusion. Human studies published in English between 1997 and 2019 were included. This time frame was chosen to capture the most recent advances of VR technology in health practice.

### Review Registration and Approach

The inclusion criteria, methods, and analysis were registered in the Joanna Briggs Institute (JBI) Systematic Review Register, and the protocol was registered with the International Prospective Register of Systematic Reviews (PROSPERO, CRD42019143504) a priori to the commencement of data extraction. The systematic review was conducted in accordance with the JBI methodology for systematic reviews of effectiveness evidence. This review followed the preferred reporting items for systematic reviews and meta-analyses (PRISMA) recommendations for systematic review reporting [49].

#### Search Strategy

To identify published studies, a 3-step search strategy was employed between April and June 2019. An initial limited search of the Medical Literature Analysis and Retrieval System Online (MEDLINE, using EbscoHost) and Google Scholar was undertaken to identify articles on the topic, using search terms “virtual reality,” “older adult*,” “community setting,” “health,” and “wellbeing.” The text words contained in the titles and abstracts of relevant articles and the index terms used to describe the articles were used to develop a full search strategy for MEDLINE, Cyberpsychology and Behavior, Cumulative Index of Nursing and Allied Health Literature (CINAHL), Exerpta Medica DataBASE Guide (EMBASE), Web of Science, JBI, the Cochrane Library, IEEExplore, Lancet Psychiatry, Association for Computing Machinery (ACM) Digital Library, Science Direct, and PsycINFO. The search strategy, including all identified keywords and index terms, was adapted for each database to account for appropriate Medical Subject Headings (MeSH) terms. Multimedia Appendix 1 shows the search strategies used for each database, with concepts combined with Boolean operators *AND* and *OR*. Hand searches were conducted when necessary. The reference list of all relevant systematic reviews was screened for additional studies. Multimedia Appendix 2 illustrates the PRISMA flow diagram.

#### Study Selection

Following the search, all identified citations were collated and uploaded into Endnote X9, and duplicates were removed [50]. Titles and abstracts were screened by 2 independent reviewers for assessment against the inclusion criteria for the review (GD and CG). The full text of selected citations was assessed in detail against the inclusion criteria by at least two independent reviewers (GD, LW, GW, and CG). Reasons for exclusion of full-text studies were as follows: participants in the intervention group being younger than 60 years, VR apps not utilizing HMDs, studies using qualitative designs, and not addressing the effectiveness of VR apps. Disagreements arising between the reviewers at any stage of the study selection process were resolved through team discussions or with a third reviewer.

#### Assessment of Methodological Quality and Certainty of the Findings

Eligible studies were critically appraised and methodological quality was assessed by 2 independent reviewers (GD, LW, GW, and CG), using the standardized critical appraisal instruments from the JBI [51]. This allowed the reviewers to gain greater insight into the methodological strengths and limitations of the selected studies. Blinding treatment groups was not always conceivable given the nature of the intervention; hence, it was not considered a criterion for inclusion. Any incongruities in appraisal that arose between reviewers were discussed and resolved by all authors. The author of one of the studies considered for inclusion in this review was contacted to request clarification of the randomization techniques used.

The grading of recommendations, assessment, development, and evaluation (GRADE) approach for assessing the certainty of evidence for an effect, summarized in a narrative form, was used to assess the overall quality of the findings [52]. The GRADE assessment evaluates the limitations of the studies, indirectness, imprecision, inconsistency, and publication bias [52]. The overall quality of the evidence was categorized as high, moderate, low, or very low. At least two reviewers (GD, LW, GW, and CG) independently completed the GRADE assessments for each article.

### Data Extraction

Data were extracted from studies by 2 independent reviewers (GD, LW, GW, and CG) using an adapted version of the JBI standardized data extraction tool. The data extracted included specific details about the populations; study methods; interventions; outcomes of significance to the review objective, including the effectiveness of the VR intervention on physical, mental, and psychosocial outcomes; types of VR apps used; and specific health outcomes targeted in community-dwelling older adults. Disagreements between the reviewers were resolved through team discussions or with a third reviewer.

### Data Synthesis

Findings from the selected studies were narratively synthesized to demonstrate the effectiveness of the VR app intervention via immersive headsets on physical, mental, or psychosocial outcomes.

## Results

### Summary of the Search Results

The initial search (Multimedia Appendix 1) from 12 databases resulted in 416 references. Additional records were identified (n=74) through manual searching, including searching for systematic review reference lists. After duplicates were removed, titles of a total of 464 studies were assessed.

After the title search was completed, the abstracts of 216 articles were assessed for inclusion by 2 authors (GD and CG). A total of 17 full-text papers were comprehensively assessed for inclusion. Of which, 10 full-text manuscripts were excluded for reasons previously stated, and 7 manuscripts were included in this systematic review, with 2 papers reporting on the same sample. To compare data, Table 1 and Table 2 present the characteristics of the studies and the key findings extracted.

**Table 1:** Details of study design, virtual reality therapy, and health outcomes.

Study	Study design	Sample size	Sample characteristics	Virtual reality application	Virtual reality exposure	Outcomes assessed	Outcomes measured
Benham [53]	Mixed methods exploratory study	12	8 female, 4 male, majority of the sample were white, majority had exposure to technology weekly, 8 participants had musculoskeletal pain, and 4 had neurological pain	The immersive VR^a^ system utilized the HTC^b^ Vive HMD^c^ with 2 hand controllers. A variety of games could be played, although those using movements or standing were excluded for safety reasons. Popular games included interactions with animals, music, and travel. Used range of commercially available VR apps	Participants completed 12 VR sessions over a 6-week period. These ranged from 15 to 45 min long.	Pain management, QoL^d^, depression, overall physical health, overall psychological health, and social life	Patient-reported outcomes, measurement information system, Item Bank v. 1.0–emotional distress–depression, World Health Organization Quality of Life Scale Brief Version (assessing overall health, physical, psychological, and social health), numeric pain rating scale
Gago [54]	Quasi-experimental	39	20 participants with AD^e^ (11 fallers and 9 nonfallers) and 19 control participants. Groups were equally matched for demographic and anthropometric data.	Immersive Oculus Rift VR headset showed a view as if the person was standing at the top of a staircase. The perspective in the headset was shifted forward down the stairs to mimic the displacement during a fall. Used the commercially released “Tuscan Villa” demo from Unity, which is no longer available	One session with a total of five 10-second trials per subject, with each trial involving 1 shift in perspective down the stairs (approximate distance of 1.17 m)	Posture and falls in AD	Kinematic and time-frequency distribution sensor data to assess posture and falls
Levy [55]	RCT^f^	16	10 women and 6 men, randomly assigned to a treatment (6 women and 3 men) and control (4 women and 3 men) group. Their fear of falling developed after falling themselves.	Immersive V8 VR HMD was used with wireless mouse for exposure therapy, followed by the use of serious games. Participants were asked to walk during the VR exposure in different settings on different difficulty levels (city, castle, island, and underground). A 3-dimensional electromagnetic sensor was fixed to the helmet to measure head and body motion during walking periods. Used bespoke research software.	12 weekly sessions lasting 40 min (VR exposure=15 min)	Falls, depression, anxiety, and social life impact from functional impairment	Fear of falling measure, Beck depression inventory, Spielberger state-trait anxiety inventory, and Sheehan disability scale
Optale [56]	RCT	36	24 female and 12 male; from local rest-care home	Compare face-to-face music therapy (control) with VR therapy that includes the music from control condition. Custom virtual environments including outdoor areas with paths, childhood homes, or modern city. Navigated using joystick and viewed through motion-tracked V6 headset. User navigates through the environment and views video clips at certain points. Music plays during the experience. Used bespoke research software.	The experimental group completed 6 months of VR memory training. 3 VR sessions every fortnight within the first 3 months. During the next 3-month booster training phase, there was 1 weekly VR session. VR sessions lasted approximately 15 min.	General cognitive ability and memory, depression, and daily living	General cognitive abilities were assessed using the mini-mental state examination and mental status in neurology tests; the digital span test assessed short-term verbal memory abilities; verbal story recall test assessed long-term verbal memory; phonemic verbal fluency test, dual task performance test, and cognitive estimation test were used to assess executive function; clock drawing test assessed visuospatial processing; activities of daily living functions and mobility and the instrumental activities of daily living assessed daily living activities; the geriatric depression scale assessed depression.
Parijat [57,58]	RCT	24	12 male and 12 female; 12 control and 12 experimental; closely age matched (±4 years); closely matched for physical characteristics.	Participants viewed a custom city-street VR environment that moved as if the person was walking, through an immersive motion-tracked Sony headset. The visual scene showed that the person was slipping, to induce slip recovery behavior. Used bespoke research software.	One session of VR exposure of 45 to 55 min, with pre- and postslip training and assessments in separate sessions.	Falls	Kinematic and kinetic data were filtered before angular kinematics and muscle activations were assessed at 5-min time intervals.
White [59]	Case study	1	74-year-old male living at home with his wife. Diagnosed with mild cognitive impairment with probable development of AD. Scored 24/30 on Montreal Cognitive Assessment.	Bespoke 3-story virtual building that is navigated via a wheelchair in a 2-dimensional physical space and viewed through oculus Rift DK2. Virtual elevators move the user between floors. Task involves moving to the correct window of 12 in the building. Used bespoke research software.	3×45-min training sessions per week for 7 weeks	Cognitive ability in AD	Cognitive ability and spatial trajectories were assessed

^a^VR: virtual reality.

^b^HTC: high-tech computer.

^c^HMD: head-mounted display.

^d^QoL: quality of life.

^e^AD: Alzheimer disease.

^f^RCT: randomized controlled trial.

**Table 2:** Summary of findings table.

Outcomes assessed; study	Key findings	Grading of recommendations, assessment, development, and evaluation^a^
Pain management; Benham [53]	Between presession 1 and postsession 12 VR^b^ sessions, there were significant improvements in pain scores with a large effect size (−1.54, 95% CI −2.50 to −0.58; *P*=.002; Effect size >0.8=large effect).	Moderate
Quality of life; Benham [53]	WHOQOL-BREF^c^ did not find any significant differences over 6 weeks of VR therapy on overall health (−0.06, 95% CI −0.91 to 0.78; *P*=.66), no significant differences on overall physical health (0.41, −0.45 to 1.26; *P*=.08), no significant differences on social life (0.08, 95% CI −0.77 to 0.93; *P*=.87), and no significant differences on overall psychological health (0.33, 95% CI −0.52 to 1.18; *P*=.15).	Moderate
Posture; Gago [54]	The AD^d^ faller group had a higher power regarding use of mechanical properties of oscillation for postural adjustments compared with the control group, alluding to worse postural stability in this group (−4 to 0s: *P*=.02; 0 to 4s: *P*=.01; and 4 to 8s: *P*=.008). AD participants had a time lag in cognitive strategies for postural correction compared with healthy subjects (−4 to 0s: *P*=.002; 0 to 4s: *P*=.01).	Moderate
Falls; Gago [54]	The AD fallers groups had a delayed reaction time for changes in power compared with the control group, with a change in power seen only in the last interval (0 to 4s vs 4 to 8s; LB^e^ *P*=.008; HB^f^ *P*=.01).	Moderate
Falls; Levy [55]	There were statistically significant differences between the 2 groups for fear of falling scores. Fear of falling scores over the 12 weeks reduced by 2.78 (SD 4.82) in the VR group and increased by 4.14 (SD 4.30) in the control group (*P*=.007).	Moderate
Falls; Parijat [57,58]	VR training led to significantly better balance on slippery surfaces with VR therapy reducing slip distance (slip distance 1: *F*_1,18_=10.34, *P*=.01; slip distance 2: *F*_1,18_=5.27, *P*=.03), reducing peak slide heel velocity (*F*_1,18_=4.54, *P*=.05), and reducing peak trunk extension post slip (*F*_1,18_=12.46, *P*=.01). Slip distance 1 and 2 are the anterior-posterior distance traveled (in cm, based on the location of the heel) from the start of the slip to when heel acceleration peaks (slip distance 1) and then from this point until the heel velocity peaks (distance 2) VR with the treadmill supported realistic walking gait after 15 to 20 min (step duration: *F*_6,76_=10.56, *P*=.002; step width: *F*_6,76_=9.56, *P*=.02). There were no significant effects on ankle, hip, or knee kinematics.	Moderate
Memory and cognitive function; Optale [56]	Combining music therapy with exploration of spatial and personally relevant environments in VR led to improved memory (*F*_2,58_=17.40; *P*<.001) and general cognitive functions (MMSE^g^ scores *F*_2,58_=23.01, *P*<.001; mental status in neurology score *F*_2,58_=30.16, *P*<.001); executive function (cognitive estimation test group difference: *F*_1,29_=11.12, *P*=.002; dual task performance test group and time interaction: *F*_2,58_=10.92, *P*<.001; phonemic verbal fluency test group and time interaction: *F*_2,58_=14.6, *P*<.001); verbal memory (digital span test group and time interaction *F*_2,58_=17.4, *P*<.001; verbal story recall test group and time interaction *F*_2,58_=36.66, *P*<.001), but not in spatial abilities (*F*_2,58_=3.14, *P*=.05).	Low
Memory and cognitive function; White [59]	Navigation errors reduced during VR training, but there may be no strong positive effect on overall cognitive ability.	Low
Disability; Levy [55]	There were no significant differences in social life (*P*=.18) or family life (*P*=.12) impact from functional impairment between the VR exposure therapy group and the waiting-list group.	Moderate
Daily living; Optale [56]	There were no significant differences of VR therapy on daily living tasks (Activities of Daily living _2,58_=1.5, *P*=.23; Instrumental Activities of Daily Living *F*_2,58_=1.05, *P*=.36).	Moderate
Anxiety; Levy [55]	There were statistically significant differences between the 2 groups’ mean state anxiety scores. The mean state anxiety score reduced by 8.86 (SD 14.46) in the VR group and increased by 9.80 (4.66) in the control group (*P*=.005). There were no significant differences in trait anxiety scores between the groups (*P*=.24).	Moderate
Depression; Benham [53]	There were no significant differences in PROMIS^h^ scores (0.29, 95% CI −1.14 to 0.56; *P*=.33).	Moderate
Depression; Levy [55]	There were no significant differences in Beck Depression Inventory scores between the VR and control groups (*P*=.47).	Moderate
Depression; Optale [56]	The participants receiving VR therapy had a reduced depression value on the Geriatric Depression Scale after the initial VR session (*F*_1,29_=5.61; *P*=.02), but not after the booster VR session (*F*_1,29_=1.35; *P*=.25).	Moderate

^a^GRADE assessment reported per outcome, not per study.

^b^VR: virtual reality.

^c^WHOQOL-BREF: World Health Organization Quality of Life Scale Brief Version.

^d^AD: Alzheimer disease.

^e^LB: low-frequency band within kinematic time-frequency analysis.

^f^HB: high-frequency band within kinematic time-frequency analysis.

^g^MMSE: mini-mental state examination.

^h^PROMIS: patient-reported outcomes measurement information system.

### Populations Included in the Review

The populations recruited were highly variable. Although all were 60 years and older, the populations recruited into the studies in this review were older adults experiencing musculoskeletal or neurological pain, with a fear of falling, living in residential aged care, diagnosed with AD, and diagnosed with mild cognitive impairment [53-59]. Therefore, the results could not be synthesized across defined populations, for example, older adults at risk of falls.

### Virtual Reality Application

VR apps were used to view a stereoscopic virtual environment through a motion-tracked HMD. Each of the studies in this review used an immersive virtual environment intervention [53-59]. This included differently manufactured devices with varying frames per second, field of view, degrees of freedom, latency, and tracking, which influence the immersive sense of presence [19]. The frequency and duration of VR exposure varied considerably between studies. VR exposure ranged from five 10-second displays of stereoscopic 3D images to 55 min of serious gaming, with 15 min being the most common exposure time [53-59]. VR therapy was administered as a singular session in 3 studies [54,57,58]. Other studies completed VR therapy more regularly, with Levy [55] administering it weekly over a 12-week period, Benham [53] completing it twice weekly for 6 weeks, White [59] completing it 3 times per week over a 7-week period, and Optale [56] completing 3 sessions per fortnight. Across the 7 papers, VR was used for 2 general categories of health care application: *treatment* for a particular condition or symptom and *assessment* of physical ability (although there was also a component of training) [53-59]. A total of 2 papers used VR apps that were commercially available at the time: Benham [53] allowed participants to use apps from VivePort, an web-based software portal (www.viveport.com), whereas Gago [54] made use of the *Tuscan Villa* demonstration environment that was provided in the Unity game engine software (which is no longer available) [20]. All other papers utilized bespoke software designed and created for the purposes of the research.

The papers in the review shared few commonalities, as a number of different health outcomes were addressed and a range of VR headsets and apps were utilized. Despite this, VR was often found to be effective or beneficial in addressing health in older adults, as it has with pain and physical activity [53-59]. This suggests that it can be a flexible and effective means of intervention delivery.

### Health Outcomes Targeted in the Interventions

#### Physical Health Outcomes

Physical health outcomes are important for community-dwelling older adults to remain living in the community as independently as possible. However, due to the complex multimorbid chronic disease, many community-dwelling older people are at risk of declining physical health as they age [60,61]. For example, older adults living in the community are at greater risk for falls due to increasing functional decline and subsequent muscle loss and weakness. Several studies focused on falls, including participants who were either at risk of falls or with a history of falls [54,55,57,58]. Some overlap in health outcomes existed where 2 studies focused on health outcomes that transect physical, mental, and social aspects [53,55]. For example, the paper by Levy [55] had a primary objective focused on assessing VR therapy in the treatment of fear of falling. In total, 2 studies evaluated the use of VR training apps to improve postural and muscular adjustment with the goal of enabling the older person to compensate for induced perturbation (balance-recovery) or slips with the goal of fall prevention [54,57]. In perturbation-balance training, a balance-recovery approach is used to improve the recovery reaction to a person losing his or her balance. Gago [54] developed VR training that introduced visual perturbation with the goal of studying the postural adjustment mechanisms in participants with AD with and without a history of falls and a control group. A key finding of this study was that older adults with AD who had a history of falling needed greater compensatory postural adjustment compared with participants with AD who did not have a history of falling and the control group (−4 to 0s: *P*=.02; 0 to 4s: *P*=.01; and 4 to 8s: *P*=.008) [54]. This may be due to visual and perceptual problems that are often common in people with AD. However, the participants with AD who had a history of falls were able to make adjustments to visual perturbation [54].

Parijat et al [57,58] conducted 2 separate studies using the same sample and dataset. In the study by Parijat et al [58], the design and effectiveness of VR training to improve recovery reactions of healthy older adults, with the aim of reducing fall frequency, was investigated. Parijat et al [58] found that VR training of motor skills to recover from a slip-induced fall led to significantly better balance minutes (step duration: *F*_6,76_=10.56, *P*=.002; step width: *F*_6,76_= 9.56, *P*=.02) and recovery reactions (slip distance 1: *F*_1,18_=10.34, *P*=.01; slip distance 2: *F*_1,18_=5.27, *P*=.03; peak slide heel velocity: *F*1,18=4.54, *P*=.05) on actual slippery surfaces. However, because participants were able to adapt to virtual slips fairly quickly, visually inducing physical responses may not be effective long term. In the paper, slip distances 1 and 2 are the anterior-posterior distance traveled (in cm, based on the location of the heel) from the start of the slip to when heel acceleration peaks (slip distance 1) and then from this point until the heel velocity peaks (slip distance 2) [58]. In the study by Parijat et al [57], the objective was to improve the recovery reaction and improve angular and muscular responses when participants were exposed to a slippery surface. The authors also quantified the kinematics of angular and muscular changes [57]. Kinematics is a way of describing the geometric mechanics of motion and velocity without considering the force that caused the motion. The key findings of this study were that VR with the treadmill supported both realistic walking gait and significant improvements in slip recovery kinematics [57]. The authors concluded that slip training was more effective using VR training compared with conventional movable platform training [57]. The kinematic angular differences varied only in the trunk measurements between the 2 groups. Peak trunk extensions decreased more in the VR training group compared with the control group (*F*_1,18_=12.46; *P*=.01) [57]. In the second slip trial, the VR training group was able to quickly reverse the forward trunk rotation, which is a key ability for regaining balance [57].

#### Mental and Psychosocial Health Outcomes

A total of 3 studies implemented VR to improve cognition, memory, and/or psychologic aspects in older adults [55,56,59]. In the study by Levy [55], older adult participants who exhibited fear of falling were included. The objective of this study was to use a VR app to treat the pathology of the phobic reactions to walking experienced by the participants [55]. Notably, all participants had moderate to severe social limitations as a result of their traumatic fall history, in addition to having a variety of comorbidities that were not related to walking difficulties [55]. The findings of this study showed a significant decrease in the fear of falling measure in the intervention group (−2.78; SD 4.82) compared with the control group after VR exposure (4.14; SD 4.30; *P*=.007) [55]. Although there was no difference in social life or depression, anxiety in the intervention group was significantly lower (−8.86; SD 14.46) than that in the control group (9.80; SD 4.66; *P*=.005) [55]. Optale [56] and White [59] studied the use of VR apps to improve cognition and memory function in older adults with memory deficits. Optale [56] used VR immersion and interaction as a treatment intervention for older adults with memory deficits. Several outcomes were measured, including general cognitive abilities, verbal memory, executive functions, and visuospatial processing. The study findings show that VR therapy led to significant improvements in cognitive functioning and verbal memory (pretraining to posttraining MMSE score: *F*_1,29_=6.85; *P*=.01 and posttraining to postbooster evaluation: *F*_1,29_=4.46; *P*=.04), particularly long-term memory improved in the intervention group [56]. A significant change was observed in the experimental group for all 3 executive functions measured [56]. However, visuospatial abilities or daily living were not affected [56]. A descriptive case study by White [59] used VR app intervention to strengthen the cognitive reserve in one participant with early AD to improve and maintain cognition, and in particular, spatial cognition. The participant’s navigational ability improved in response to the training given [59]. Although navigation errors were present, a reduction was evident overall [59]. However, it is unclear whether or not there is a positive effect on overall cognitive ability [59]. The findings of this case study suggest that people with early stages of AD can learn to navigate paths in a suitable immersive VR system [59].

In the remaining study, Benham [53] conducted a mixed methods study at a senior day-center to identify the efficacy of VR interventions on pain, depression, and QoL in community-dwelling older adults who self-reported acute or chronic pain that was bothersome at least two days per week. The VR therapy appears to have improved pain management in community-dwelling older adults [53]. VR intervention significantly reduced the report of pain (−1.54, 95% CI−2.50 to −0.58; *P*=.002) [53]. Participants also reported that the VR intervention was able to distract them from their pain [53].

## Discussion

### Principal Findings

The incorporation of VR in health care is promising in its ability to support older adults in managing age-related changes, for example, musculoskeletal changes and those related to chronic disease that impact physical, cognitive, and psychosocial health and well-being. The studies included in this review are examples of how technologic advances are changing the face of health care and demonstrate that certain VR interventions can be successfully used with older adults.

Fall prevention appears to be an area where immersive VR using HMD can have a significant impact. Falls contribute to injury, pain, disability, and premature death [5]. The loss of balance and slow reactions to steady state is a primary mechanism for falls [62]. A total of 2 studies evaluated the use of VR training apps to improve postural and muscular adjustment with the goal of enabling the older person to compensate for induced perturbation (balance-recovery) or slips so that a fall may be prevented [54,57]. Although there is an emphasis on the physical aspect of fall prevention, including balance, gait, and recovery reaction, sustaining a fall can have psychological and behavioral impacts on the older person. Fear of falling is common among older adults, and in some cases, this fear is disproportionate, with physical reactions to extreme anxiety including palpitations, sweating, and avoidance of walking altogether [63,64]. A total of 3 studies aimed to improve cognition, memory, and/or psychological aspects in older adults [55,56,59]. The results indicate a positive change and improvement, although several questions remain around the longevity of the impact [55,56,59].

The findings of the studies reviewed suggest that the expansion of VR for health-related interventions, while in a safe and controlled environment, may have several potential therapeutic benefits for older adults to facilitate independence and QoL [65,66]. Although the studies included in this review have mostly focused on evaluating the effectiveness of VR intervention in conventional therapy [53-59], VR intervention apps could also support therapists and clinicians to assess a person’s physical, cognitive, or psychosocial status to identify potential problems [67,68]. For example, gait patterns, postural adjustment, musculoskeletal pain, and compensatory mechanisms could be assessed using VR to identify mobility issues of concern and determine a person’s fall risk [69]. Tailored VR interventions could be developed to target fall prevention and to promote increased physical activity, which has a protective effect on age-related musculoskeletal changes [54,57,58].

Furthermore, using VR to assess, develop, and implement cognitive interventions to improve cognition, memory, and psychological aspects could make VR a competitor to traditional cognitive rehabilitation [70]. For example, stroke is common among older adults, and many require cognitive rehabilitation along with physical rehabilitation. Clinicians could use VR as an individualized intervention to determine cognitive and decision-making problems and develop individualized VR-based interventions [70,71]. Chronic pain is common in older adults, limiting independence, physical movement, and social activity [53,72]. Although more knowledge is needed about VR interventions to reduce or manage pain in older adults, VR has been used extensively in other age groups, and there is scientific evidence that VR can reduce the pain that is experienced [73,74]. VR exposure therapy interventions have been applied to reduce extreme fears such as phobias [73-75]. After a fall or similar accident, older adults often suffer from extreme fear of falling, which could be very limiting to staying physically active [76]. The study by Levy [55] was able to demonstrate significant reductions in the fear of falling. One of the unique benefits of VR use in health care with older adults is the potential to meet more than one need, as VR apps may span the physical, mental, and psychosocial needs of a person [77,78].

Although some studies under review reported indication of positive outcomes regarding VR use in older adults [53,55,59], it is important to highlight several practical aspects that are challenges to implementing VR interventions with older adults, who are often in a vulnerable state, and to translate the use of VR interventions as a standard of care in the aged care sector. The majority of participants were healthy enough to fully participate in the intervention studies [57-59]. Investigations into using VR interventions with older adults who are frail or unwell are needed. For example, a better understanding of the severity of cognitive impairment and the applicability of VR has not been explored. People with increased levels of frailty who are at greater risk for functional decline and falls may benefit from VR interventions; however, it is unclear whether frail older adults can engage with the VR interventions described in this review.

### Strengths and Limitations

Visual and auditory changes could make it challenging for some older adults to engage with the technology. It is important to consider natural age-related changes in the design of VR apps for older adults [79]. Only Parijat et al [57,58] explicitly assessed visual acuity, via Snellen’s chart; Benham [53], Gago [54], Levy [55], and Optale [56] excluded participants if they self-reported uncorrected vision or serious/significant sensory impairment. None of the authors administered hearing tests [53-59]. Only Benham [53] and Parijat et al [57,58] took any measurement of cybersickness [80]. In most of the studies, it was unclear whether the researchers used a participatory approach by inviting older adults to contribute to the design and conduct of the intervention [81]. Although some literature indicates that older adults are open to using VR interventions, not much is known about the acceptance of HMD VR use in older adults [66,82]. For example, only 1 study included a survey question about the overall experience with VR [53]. None of the other studies discussed how well older adults accepted the VR and HMD and whether they were able to engage with and adapt to the physical aspects of the VR device and the VR environment or their experience with *presence*. Unless there is some adaptation to vision and hearing abnormalities and dexterity, older adults may be reluctant to adopt VR as a consistent health care–related intervention. In using VR exposure therapy with older people with anxiety and phobias, there is some debate on the outcome expectancy of treatment outcomes induced by *presence* (or immersion in the VR environment) because this relationship could be influenced by anxiety that may be inadvertently created by *presence* [55]. Subsequently, clinicians and industry providers may be reluctant to adopt VR for exposure therapy.

We noted that several studies in this review used a VR intervention as a means to impact another outcome that was not measured at the time of the study [54,57,58]. For example, although some of the studies implemented VR training with the aim of preventing falls, fall prevention was an indirect outcome variable, and although postural adjustment may have improved during the study, it is unclear how long this benefit lasts for and whether any longer-term impact on falls was experienced. Furthermore, some authors noted that VR interventions that induce slips need to make each slip novel in some way to reduce the likelihood that participants adapt to the stimulus and inadvertently limit the desired postural improvement [57,58].

Interestingly, no study designed or incorporated gaming technology in VR to support engagement and promote enjoyment for participants [26]. This would bring the technology *full circle* with its beginnings as a gaming technology [83]. The participants in the study by Benham [53] were able to play a game if they desired, but many seemed to choose other activities such as traveling or interacting with animals, and gaming/gamification was not a deliberate or controlled part of the intervention design or evaluation. Longitudinal studies should be conducted to determine the long-term effect on the outcome variable under study. All studies in this review included a small sample size, which impacted the generalizability of the findings [53-59]. Powered RCTs are needed to determine the effectiveness of VR therapy compared with standard care practices.

It is important to note that none of the papers included in the review reached a *High* level of GRADE certainty and quality. White [59] was judged to have a very low GRADE judgment, as it constituted a single case study with no blinding or randomization. The remaining papers were rated as *Moderate*, as they employed randomized designs, blinding, patient-reported outcomes, or RCTs [53-58]. Therefore, there is convincing evidence that VR interventions can be effective; however, it is clear that more research needs to be done. A recent review paper highlighted a similar shortcoming in scientific rigor in other fields of VR research [84]. So, although this suggests that it is not a problem particular to the health domain, it also emphasizes that more rigorous scientific methods need to be used to robustly evaluate and validate the technology.

### Conclusions

In conclusion, the wider literature suggests that VR interventions have the potential for wide-scale adoption to promote health and wellness among older adults. This review demonstrates that interventional research using VR with older adults in varying states of health is in the early stages of development. In particular, evidence around the potential adoption of VR interventions in applied clinical or therapeutic settings is limited and requires further understanding of logistics, financial costs, and the acceptability and usability of immersive VR in older adults from the perspective of older adults and clinicians. The review also indicated the need for a greater understanding of the design features of immersive VR apps that can promote and improve health for older adults, including those with audio-visual deficits.

## Appendix

Multimedia Appendix 1 Search strategy.

Multimedia Appendix 2 Preferred reporting items for systematic reviews and meta-analyses flow diagram.

## Abbreviations

AD: Alzheimer disease
GRADE: grading of recommendations, assessment, development, and evaluation
HMD: head-mounted display
JBI: Joanna Briggs Institute
MEDLINE: Medical Literature Analysis and Retrieval System Online
PRISMA: preferred reporting items for systematic reviews and meta-analyses
QoL: quality of life
RCTs: randomized controlled trials
VR: virtual reality
