# Supplementary material for: The Role of Virtual Reality in Improving Health Outcomes for Community-Dwelling Older Adults: Systematic Review
Source: J Med Internet Res. 2020 Jun 1;22(6):e17331. doi: 10.2196/17331 (PMC7296414; doi:10.2196/17331)
Supplement: Multimedia Appendix 1 [file jmir_v22i6e17331_app1.docx]

**CINAHL (filter added for full text, human and English)**

((virtual reality) OR (virtual reality exposure therapy) OR VRML OR (virtual reality technolog*) OR (virtual reality headset*) OR "hmd" OR ("head mounted display") OR (head mount display) OR oculus OR (HTC Vive) OR (gear VR) OR VR) AND ((lonliness OR isolation OR social isolation) OR (physical health OR physical wellbeing) OR (physical therapy) OR (mental health) OR (mental illness) OR (mental wellbeing) OR (emotional wellbeing) OR (emotional health) OR (emotional wellness) OR (emotional therapy) OR (emotional self-care) OR (emotional regulation) OR happiness OR stress OR (experiences OR perceptions OR attitudes OR views)) AND ((community setting*) OR (community environment*) OR (community networks) OR (community health service*) OR (long term use) OR (long term care)) AND (elderly OR (older adult*) OR geriatric* OR senior* OR (older adults OR elderly OR seniors OR geriatrics))

**Medline (filter added for full text, human and English)**

((virtual reality) OR (virtual reality exposure therapy) OR VRML OR (virtual reality technolog*) OR (virtual reality headset*) OR "hmd" OR ("head mounted display") OR (head mount display) OR oculus OR (HTC Vive) OR (gear VR) OR VR) AND ((lonliness OR isolation OR social isolation) OR (physical health OR physical wellbeing) OR (physical therapy) OR (mental health) OR (mental illness) OR (mental wellbeing) OR (emotional wellbeing) OR (emotional health) OR (emotional wellness) OR (emotional therapy) OR (emotional self-care) OR (emotional regulation) OR happiness OR stress OR (experiences OR perceptions OR attitudes OR views)) AND ((community setting*) OR (community environment*) OR (community networks) OR (community health service*) OR (long term use) OR (long term care)) AND (elderly OR (older adult*) OR geriatric* OR senior* OR (older adults OR elderly OR seniors OR geriatrics))

**PsycINFO (filter added for full text, human and English)**

((virtual reality) OR (virtual reality exposure therapy) OR VRML OR (virtual reality technolog*) OR (virtual reality headset*) OR "hmd" OR ("head mounted display") OR (head mount display) OR oculus OR (HTC Vive) OR (gear VR) OR VR) AND ((lonliness OR isolation OR social isolation) OR (physical health OR physical wellbeing) OR (physical therapy) OR (mental health) OR (mental illness) OR (mental wellbeing) OR (emotional wellbeing) OR (emotional health) OR (emotional wellness) OR (emotional therapy) OR (emotional self-care) OR (emotional regulation) OR happiness OR stress OR (experiences OR perceptions OR attitudes OR views)) AND ((community setting*) OR (community environment*) OR (community networks) OR (community health service*) OR (long term use) OR (long term care)) AND (elderly OR (older adult*) OR geriatric* OR senior* OR (older adults OR elderly OR seniors OR geriatrics))

**Embase**

('aged'/exp OR elderly OR 'geriatrics'/exp OR 'older adult'/exp OR 'older adults'/exp OR senior) AND ('virtual reality'/exp OR 'virtual reality exposure therapy'/exp OR 'virtual reality modeling language'/exp OR vrml OR 'virtual reality technology' OR 'virtual reality headset' OR 'virtual reality headsets' OR 'vr' OR 'hmd' OR 'head mounted display' OR 'head mount display' OR 'oculus' OR 'htc vive' OR 'gear vr') AND ('loneliness'/exp OR 'isolation'/exp OR 'social isolation'/exp OR 'health'/exp OR 'physical well-being'/exp OR 'physiotherapy'/exp OR 'mental health'/exp OR 'mental disease'/exp OR 'mental wellbeing' OR 'psychological well-being'/exp OR 'emotional wellbeing'/exp OR 'emotional stability'/exp OR 'emotional therapy' OR 'emotional wellness' OR 'emotional self care' OR 'happiness'/exp OR 'stress'/exp OR 'emotional regulation'/exp OR 'experience'/exp OR 'perception'/exp OR 'attitude'/exp OR view) AND ('community setting' OR 'community environment' OR 'community care'/exp OR 'community health service' OR 'long term use' OR 'long term care'/exp) AND ([young adult]/lim OR [adult]/lim OR [middle aged]/lim OR [aged]/lim OR [very elderly]/lim) AND [humans]/lim AND [english]/lim

**Web of Science**

("virtual reality" OR "virtual reality exposure therapy" OR VRML OR "virtual reality technolog*" OR "virtual reality headset*" OR hmd OR "head mounted display" OR "head mount display" OR oculus OR "HTC Vive" OR "gear VR" OR VR) AND (lonliness OR isolation OR social isolation OR "physical health" OR "physical wellbeing" OR "physical therapy" OR "mental health" OR "mental illness" OR "mental wellbeing" OR "emotional wellbeing" OR "emotional health" OR "emotional wellness" OR "emotional therapy" OR "emotional self-care" OR "emotional regulation" OR happiness OR stress OR experiences OR perceptions OR attitudes OR views) AND ("community setting*" OR "community environment*" OR "community networks" OR "community health service*" OR "long term use" OR "long term care") AND (elderly OR "older adult*" OR geriatric* OR senior* OR "older adults" OR elderly OR seniors OR geriatrics)

**Lancet Psychiatry**

“Virtual reality” AND “older adult”

**Cyber-psychology and Behaviour**

“Virtual reality” AND “elderly”

**IEEExplore**

“Virtual reality” AND “older adult”

**Joanna Briggs Institute**

“Virtual reality”

**The Cochrane Library**

“Virtual reality” AND “older adult”

**ACM Digital Library**

(("virtual reality" OR "VR" OR "virtual reality exposure therapy" OR "VRML" OR "virtual reality technolog*" OR "virtual reality headset*" OR "hmd" OR "head mount* display" OR "oculus" OR "HTC Vive" OR "gear VR") AND ("lonliness" OR "isolation" OR "social isolation" OR "physical health" OR "physical wellbeing" OR "physical therapy" OR "mental health" OR "mental illness" OR "mental wellbeing" OR "emotional wellbeing" OR "emotional health" OR "emotional wellness" OR "emotional therapy" OR "emotional self-care" OR "emotional regulation" OR "happiness" OR "stress" OR "experience*" OR "perception*" OR "attitude*" OR "view*") AND ("community setting" OR "community environment" OR "community networks" OR "community health service*" OR "long term use" OR "long term care") AND ("elderly" OR "older adult*" OR "geriatric*" OR "senior*" OR "older adults" OR "elderly" OR "seniors"))

**Science Direct**

(("virtual reality" OR "VR") AND "community setting" AND ("health" OR "wellbeing") AND ("elderly" OR "older adult" OR "senior" OR "older people"))
