# Supplementary figures and images for: The Role of Virtual Reality in Improving Health Outcomes for Community-Dwelling Older Adults: Systematic Review
Source: J Med Internet Res. 2020 Jun 1;22(6):e17331. doi: 10.2196/17331 (PMC7296414; doi:10.2196/17331)

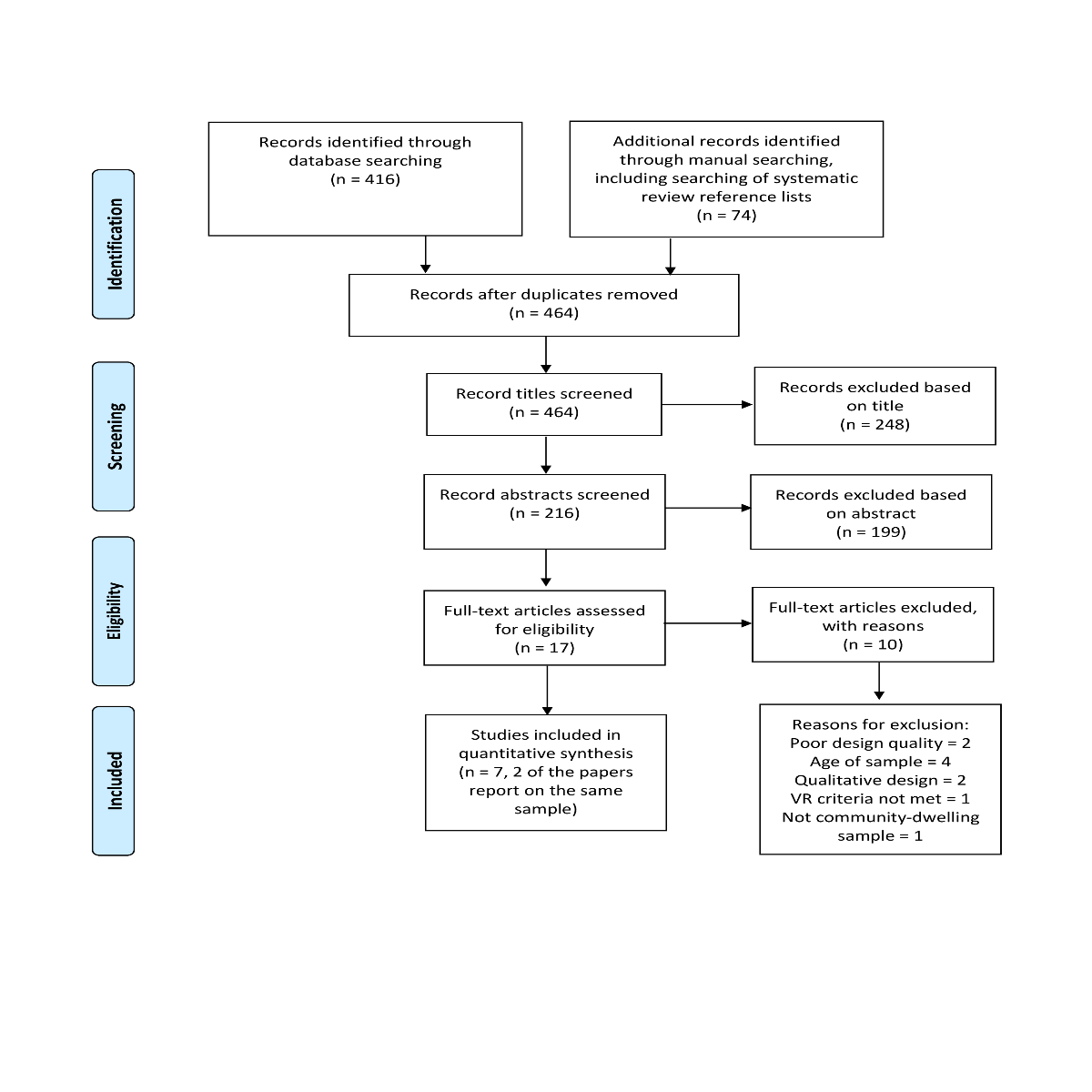

Supplement: Multimedia Appendix 2 [file jmir_v22i6e17331_app2.png]
